# Positive effects of COVID-19 lockdown on air quality of industrial cities (Ankleshwar and Vapi) of Western India

**DOI:** 10.1038/s41598-021-83393-9

**Published:** 2021-02-19

**Authors:** Ritwik Nigam, Kanvi Pandya, Alvarinho J. Luis, Raja Sengupta, Mahender Kotha

**Affiliations:** 1 School of Earth, Ocean and Atmospheric Sciences (SEOAS), Goa University, Taleigao Plateau, Goa, 403206 India; 2 Department of Geography, Faculty of Science, The Maharaja Sayajirao University of Baroda, Fatehgunj, Vadodara, 390002 India; 3 Earth System Science Organization-National Centre of Polar and Ocean Research, Ministry of Earth Science, Govt. of India, Headland Sada, Goa, 403804 India; 4 Department of Geography & McGill School of Environment, McGill University, Montreal, QC H3A0B9 Canada

**Keywords:** Atmospheric science, Climate change, Environmental sciences, Environmental social sciences, Natural hazards

## Abstract

On January 30, 2020, India recorded its first COVID-19 positive case in Kerala, which was followed by a nationwide lockdown extended in four different phases from 25th March to 31st May, 2020, and an unlock period thereafter. The lockdown has led to colossal economic loss to India; however, it has come as a respite to the environment. Utilizing the air quality index (AQI) data recorded during this adverse time, the present study is undertaken to assess the impact of lockdown on the air quality of Ankleshwar and Vapi, Gujarat, India. The AQI data obtained from the Central Pollution Control Board was assessed for four lockdown phases. We compared air quality data for the unlock phase with a coinciding period in 2019 to determine the changes in pollutant concentrations during the lockdown, analyzing daily AQI data for six pollutants (PM_10_, PM_2.5_, CO, NO_2_, O_3_, and SO_2_). A meta-analysis of continuous data was performed to determine the mean and standard deviation of each lockdown phase, and their differences were computed in percentage in comparison to 2019; along with the linear correlation analysis and linear regression analysis to determine the relationship among the air pollutants and their trend for the lockdown days. The results revealed different patterns of gradual to a rapid reduction in most of the pollutant concentrations (PM_10_, PM_2.5,_ CO, SO_2_), and an increment in ozone concentration was observed due to a drastic reduction in NO_2_ by 80.18%. Later, increases in other pollutants were also observed as the restrictions were eased during phase-4 and unlock 1. The comparison between the two cities found that factors like distance from the Arabian coast and different industrial setups played a vital role in different emission trends.

## Introduction

The novel coronavirus termed as COVID-19 by World Health Organization (WHO), first emerged in late December 2019 in Wuhan, China. In early March 2020, due to its rapid spread, the WHO declared COVID-19 as a pandemic. By July 8, 2020, it spread to more than 210 countries worldwide, infecting over 11 million people and causing 539,026 mortalities^1^. As COVID-19 is highly transmissible, along with a high mortality rate^2^, countries worldwide have taken various precautionary measures, such as large scale COVID-19 screening tests, quarantine, social distancing, wearing of mask, sanitization of hands, etc^3^. This led to 2–4 weeks of regional lockdowns to limit the spread of the virus, all of which have subsequently restricted economic activities around the world leading to different regional repercussions^4^.

**Figure 1:**
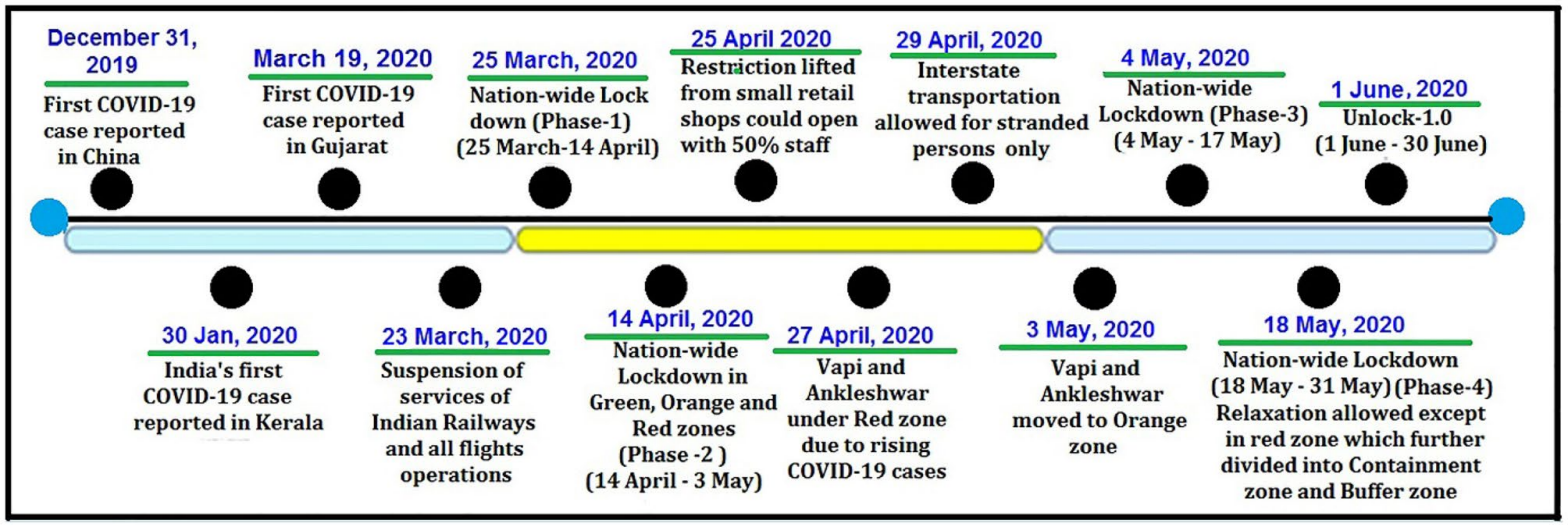
COVID-19 timeline of India. (Source: Ministry of Home Affairs, Govt. of India).

In India, a student who had returned from Wuhan, China, was the first COVID-19 positive case recorded on January 30 in Kerala^5^. India took unprecedented measures to contain the infection from across borders and within its territory. International travel and non-essential traveling visas were suspended on March 13, 2020. The Indian railways shut down its operations on March 23, 2020, for the first time in its history spanning over 167 years. A 21-day nationwide lockdown phase-1 was enforced from March 25 to April 14, which was extended further until May 31, 2020. Divided into different phases, the lockdown was marked by increasing relaxations in socio-economic activities in less infected regions. The timeline of the various COVID-19 lockdown phases in India^6^ is depicted in Fig. 1.

While the socio-economic devastation due to COVID-19 has been colossal around the world, which required "a wartime" plan from every corner of the world^7^ it has also come as the silver lining for the environment^8^. The United Nations Environment Program chief Inger Andersen believes these environmental changes are temporary^7^, as the global environment had a small respite before industrial activities resumed since February 2020. Recent studies have reported improvement in air quality due to restrictions placed upon industrial activities during the lockdown. Climate scientists have indicated that greenhouse gaseous (GHGs) concentration could drop to levels not seen since World War II. Highly industrialized cities located in cold climate zones observed a higher reduction in air pollution^9^.

Lockdown in various countries viz., France, Germany, Italy, Spain, and China led to shutting down of power plants, transportation, and other industries which resulted in drastic decrease in concentration levels of GHGs, NO_2_, PM_2.5_, PM_10_ and CO but spikes in ozone concentration simultaneously, primarily in Europe and large Chinese cities^3,10–14^. The air quality changes during COVID-19 lockdown over the Yangtze River Delta Region suggest that the reduced human activity and industrial operations lead to significant reduction in PM_2.5_, NO_2_, and SO_2_^14^. Significant improvement in air quality, as evidenced from the reduction in Particulate Matter, NOx, SO2 and CO, during the COVID19 lockdown period was observed in the Hangzhou megacity^15^. Reduction of NO_2_ (49%), and CO (37%) concentrations in the USA during lockdown were positively correlated with higher population density^3^. The impact of the measures on the air quality is discussed^16^ for the city of Rio de Janeiro, Brazil by comparing the particulate matter, carbon monoxide, nitrogen dioxide and ozone concentrations during the partial lockdown with those of the same period of 2019 and also with the weeks prior to the virus outbreak. A positive impact of the social distancing measures is reported^17^ on the concentrations of the three main primary air pollutants (PM_10_, NO_2_ and CO) of the São Paulo and Rio the Janeiro, the two most populated cities, wherein, the CO levels showed the most significant reductions (up to 100%) which was related to light-duty vehicular emissions. Changes in levels of some air pollutants due to a set of rapid and strict countermeasures limiting population's mobility and prohibiting almost all avoidable activities was evaluated in the in Salé city (North-Western Morocco)^18^. Barcelona city was assessed^19^ for air quality using a remote sensing dataset provided by ESA's Tropospheric monitoring instrument (TROPOMI) along with local air quality monitoring data to assess differences in air quality during the lockdown and one month before the lockdown. The observed reductions were 31% and 51% in NO_2_ and PM_2.5_, respectively, due to lockdown. The National Aeronautics and Space Agency (NASA), using the TROPOMI sensor, observed a reduction of 10–30% in Nitrogen Dioxide (NO_2_) in central and eastern China during early 2020^20^. 27% reduction was observed in nitrogen oxides concentration in comparison to the last five years, and non-uniform trends in O_3_ concentrations during the lockdown in California basin region^21^. Black carbon reduction due to the lockdown imposed restricted anthropogenic activities is observed in Hangzhou city of China^22^. A reduction of 43% and 31% in PM_10_ and PM_2.5_, while a 17% increment in O_3_ concentration during the lockdown period and past 4-year values for different regions of India has also been reported^23^.

The common air pollutants in cities and industrial towns are NO_2_, SO_2_, PM_10_, which are responsible for cardiovascular and respiratory diseases^24,25^. The primary sources of these pollutants are vehicular exhaust, road dust, and mainly metal processing industries^26,27^. The majority of the health benefits were observed with the reduction in NO_2_ in 31 provincial capital cities in China^12^. Continuous degradation of air quality in some of the Indian metropolitan cities (New Delhi, Mumbai, Kolkata, Chennai), that often exceed the standards set by WHO and Central Pollution Control Board (CPCB), India, cemented their regular presence in the list of top 20 polluted cities of the world^28–30^. The Ministry of Earth, Forest, and Climate change (MoEFC) under its National Clean Air Programme (NCAP) launched a five-year action plan in 2019 to reduce by 30% the nationwide concentration of particulate matter^31^. Due to the mandatory lockdown imposed across the country, 88 Indian cities have observed a drastic reduction in air pollution^23^.

Gujarat, which is the industrial state in western India, observed a significant reduction in major air pollutants between the lockdown period (March 25 to April 20, 2020) mainly due to restrictions on traffic and slowdown of production at factories^32^. According to the CPCB-AQI database, air pollution reduction occurred merely in four days since the lockdown^33^. In Vapi, PM_10_, PM_2.5_, NO_2_, SO_2_ are the major air pollutants significantly emitted by transport vehicles and industrial^34^.

This present study is undertaken to determine the differences in concentration of six pollutants (PM_10_, PM_2.5_, CO, NO_2_, O_3_, and SO_2_) during the lockdown period (March 25 to June 15, 2020) with the comparable period in 2019, to assess the impact of lockdown on air quality in cities Vapi and Ankleshwar of Gujarat, India.

## Study area

**Figure 2:**
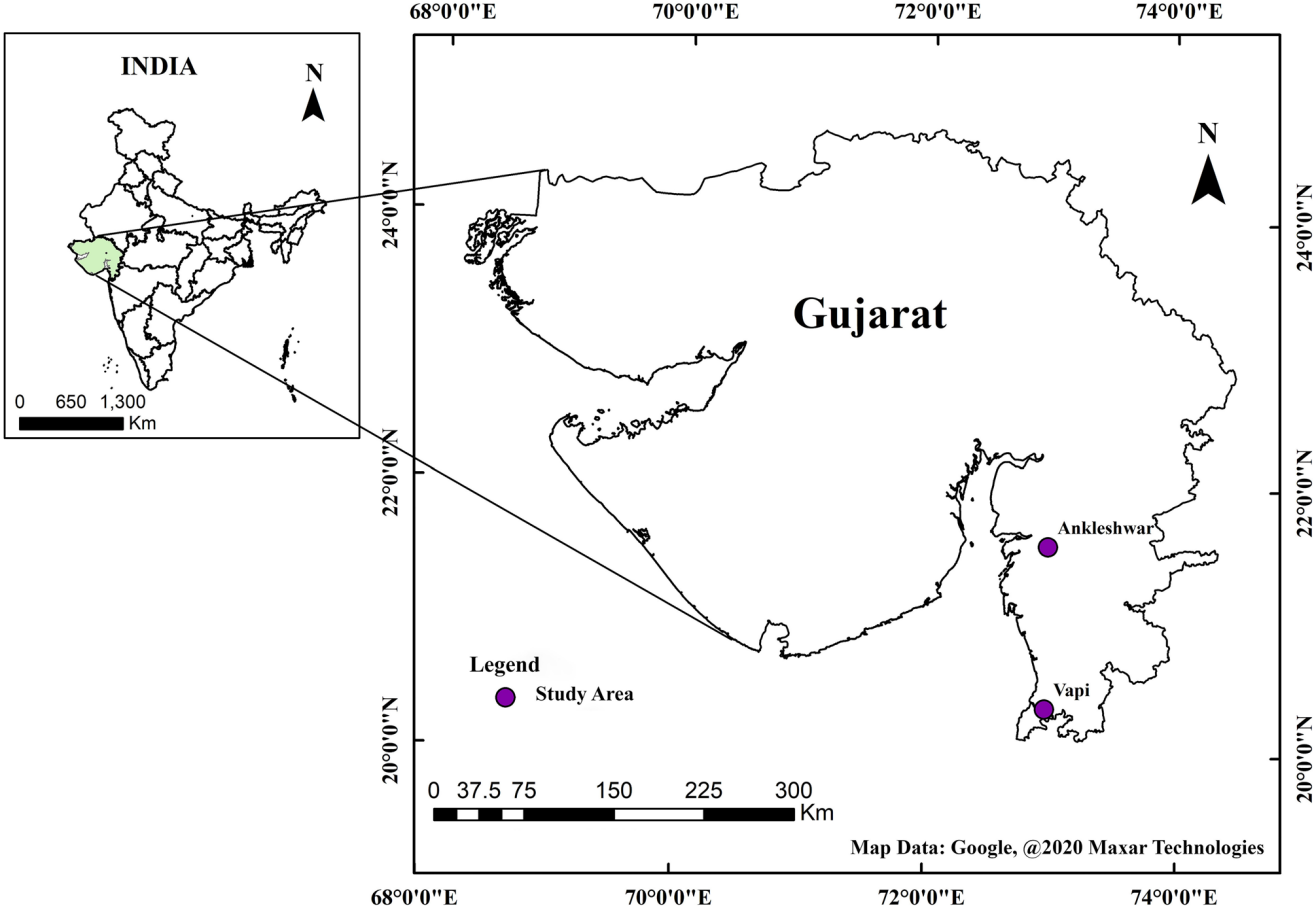
The locations of industrialized cities Ankleshwar and Vapi in Gujarat, India.

Ankleshwar is located at 21.62°N, 73.01°E is a municipality under Bharuch district juridiction in Gujarat, India (Fig. 2). It is located in the south Gujarat region in between Ahmedabad—Mumbai industrial corridor on the southern banks of lower reaches of the Narmada river. The city has plain topography with an average elevation of 15 m above mean sea level. The climate of South Gujarat region is mainly influenced by the Arabian Sea. Pre-monsoon showers announce the arrival of monsoon only in late june, with hot summer months (March to June), heavy to moderate monsoon rain (July to September), and moderate winter months (November to February). Ankleshwar Gujarat Industrial Development Corporation is spread over an area of 1600 hectares and houses more than 2000 industries with over 1500 chemical plants producing pharmaceuticals, paints, and pesticides.

Vapi, located at 20.3893°N 72.9106°E, is a municipality under the Valsad district in Gujarat, India (Fig. 2), located at the southernmost tip of Gujarat between Surat in the north and Mumbai (Maharashtra) in the South. Sandwiched between the union territories of Daman & Diu and Dadar and Nagar Haveli, the city is 7 km inland from the Arabian sea; thus, experiences coastal tropical weather with annual rainfall ranging from 100 to 120 in. starting from late June and go on till September^35^. Vapi is also a major industrial hub, 160 km south of Ankleshwar, predominantly housing chemical plants that account for 70% of the total industries in the city. Other industries are packaging, paper, plastics, and rubber. Vapi is also known as a ‘Paper hub’ as it houses the best quality Kraft paper manufacturing units in India. The city has the largest Common Effluent Treatment Plant (or CETP) in Asia^36^. However, due to ever-increasing air and water pollution, Vapi and Ankleshwar are the most industrial clusters of Gujarat (especially Vapi regularly makes it to the list of the most polluted cities in India^37^.

## Material and method

The Government of India, in 2016, under its 'Swacch Bharat Mission,' launched the 'National Air Quality Index' (NAQI)^38^. The NAQI bulletin is published daily by the Central Pollution Control Board (CPCB). There are two different techniques to monitor air quality; online monitoring network and manual monitoring network. The online monitoring network is more reliable than its counterpart as it provides pollutant concentration data almost in real-time. The automatic monitoring network AQI consists of monitoring of eight major parameters (Table 1) to compute the index value, while the manual monitoring network AQI considers mainly PM_10_, SO_2_, and NO_2_ pollutants^39^. Under the NAQI, the averaging time for pollutants such as: PM_2.5_, PM_10_, NO_2_, SO_2_, Pb, and NH_3_ is 24-h whereas, O_3_ and CO have the averaging time of 1-h. Except for CO which is measured in mg/m^3^, all other seven pollutants are measured in μg/m^3^.

**Table 1:**
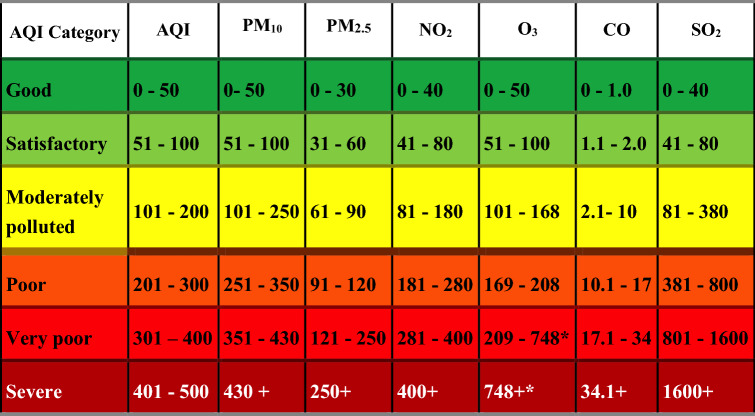
Breakpoints for AQI Scale 0–500 (all pollutants are in units of μg/m^3^ and CO is expressed in units of mg/m^3^).

*Hourly monitoring Source: Central Pollution Control Board^39^.

The breakpoint table of daily NAQI provides numeric values and color codes. The color codes are dependent on the numeric values: the AQI value between 0 and 50 suggests it as good with minimal impact on health and shown by dark green color code. Likewise, values in the range of 51–100 are termed as satisfactory (light green) wherein minor breathing discomfort occurs to sensitive people, range of 101–200 is termed as moderately polluted (yellow), range of 201–300 is termed as Poor (orange), values in the range of 301–400 are termed as very poor (light red) and, values in the range of 401–500 as listed as severe (dark red)^40^.

The objective of the NAQI is to assist with monitoring of daily ambient air quality and generate a multi-temporal database. The AQI keeps vigil on air pollutant concentration levels to determine their violation above the permissible limits of ambient air quality in the given area. 200 AQI stations are continuously monitoring air quality across the country^40^. All India AQI monitoring consists of several organizations like the CPCB, the State Pollution Control Boards (SPCB), National Environmental Engineering Research Institute (NEERI), Nagpur, and pollution control committees. The CPCB coordinates with all these agencies to ensure the uniformity, consistency of the air quality data, and provide technical and financial support to them for operating the monitoring stations^38^.

The mathematical equation for calculating sub-indices of AQI is as follows:

1 $$\mathrm{Ip}= \left(\frac{\mathrm{IHI}-\mathrm{ILO}}{\mathrm{BPHI}-\mathrm{BPLO}} \times (\mathrm{CP}-\mathrm{BPLO})\right)+\mathrm{ILO},$$

where I_P_ is AQI for pollutant “P” (Rounded to the nearest integer), C_P_ the actual ambient concentration of pollutant “P”, B_PHI_ the upper-end breakpoint concentration that is greater than or equal to C_P_, B_PLO_ the lower end breakpoint concentration that is less than or equal to C_P_, I_LO_ the sub-index or AQI value corresponding to B_PLO_, I_HI_ the sub-index or AQI value corresponding to B_PHI._

The total lockdown duration of 84 days was divided into five different periods based on different nationwide lockdown phases imposed by the Government of India to compare with 2019. The five phases of lockdown (four lockdowns and one unlock-1.0) had different sets of rules and restrictions for the general public. Phase-1 of the nationwide lockdown lasted from March 25 to April 14, 2020, with complete restrictions on economic activities. During the second lockdown phase which lasted for 19 days starting from April 15 to May 3, various parts of the cities were color-coded into green, orange, and red zones based on the number of COVID-19 positive cases; the red zones indicating rapidly rising cases had total lockdown, orange zones (moderately rising cases) were provided with some relaxation, and the green zone (low positive cases) had least restrictions among them. The third phase lasted for 14 days (May 4–17, 2020), whereas, the fourth phase (labeled as the last period of nationwide lockdown with change from the green, orange and red zones to the containment zone and buffer zone) was extended from May 18–31, 2020. The unlock 1.0 (referred to in the study as ‘phase-5’) commenced on June 1, 2020, with several restrictions uplifted everywhere, except in the containment zones. The differences in the magnitude of restrictions imposed during various lockdown phases had an indirect impact on the fluctuation in the air pollutant level due to the restarting of several economic activities in the cities.

The concentration values of six air pollutants (PM_10_, PM_2.5_, NO_2_, O_3_, CO, and SO_2_) during the nationwide lockdown period in 2020 and for the similar period of 2019 were downloaded from the daily NAQI data portal available at cpcb.nic.in which is maintained by the Ministry of Environment, Forest and Climate Change, Government of India^40^. The Meta-analysis of continuous data was performed using descriptive and inferential statistical techniques to determine the number of variations (reduction or increase) in the air pollutant levels during the different phases, to calculate the mean differences. Additionally, standard deviation was also computed to determine the fluctuation of air pollutants during different periods. Each air pollutant's mean differences during different phases for both the cities were compared to determine the impact of lockdown restrictions at two different geographical locations with similar economic characteristics. Lastly, a linear regression analysis was performed to determine the relationship of the AQI values with the different lockdown phases.

## Results

**Figure 3:**
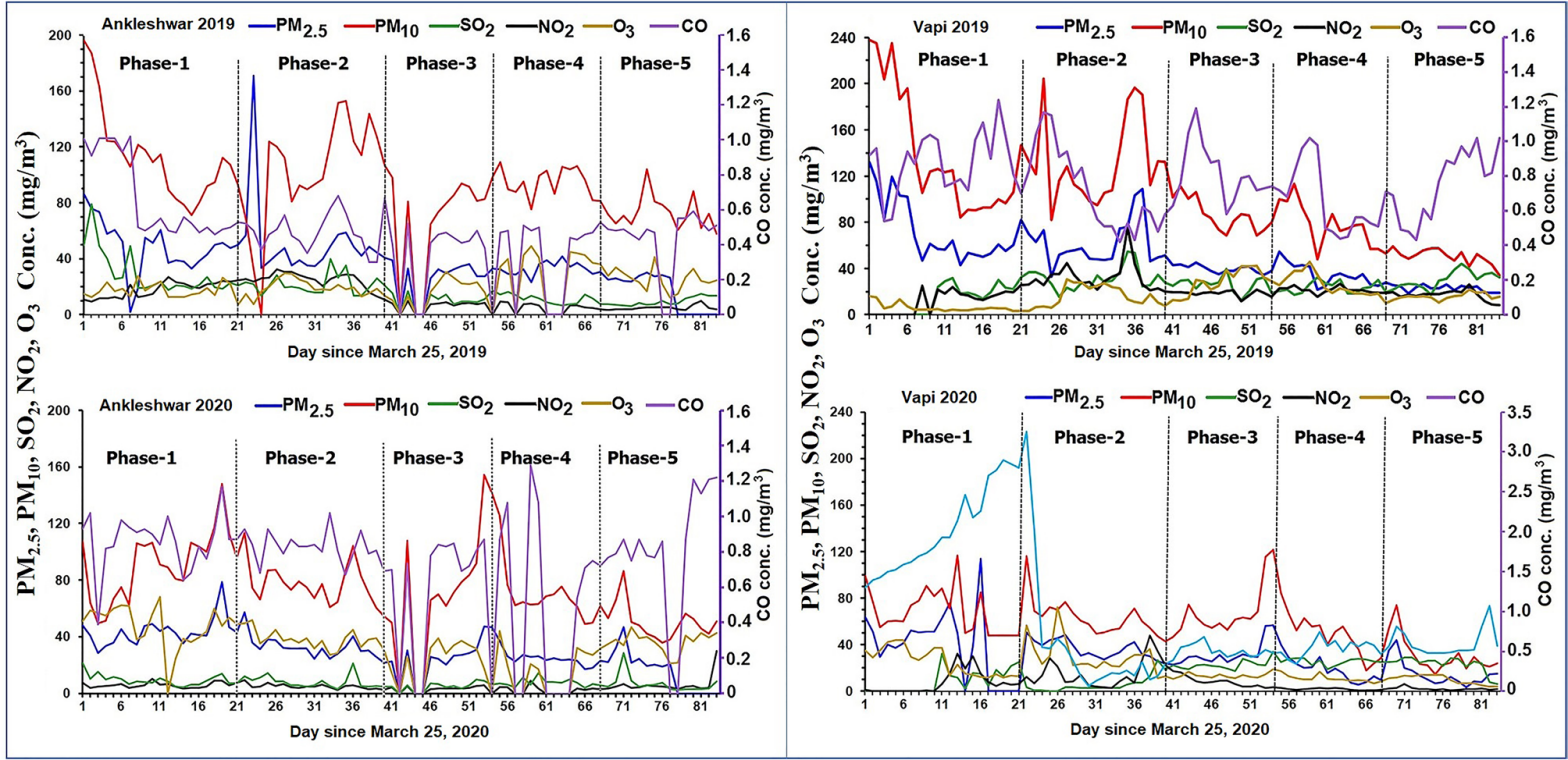
Mean concentrations of air pollutants from March 25 to June 15 for the year 2019 and 2020 for Ankleshwar and Vapi.

The mean distribution of air pollutants during lockdown for COVID-19 and a similar period in 2019 for the two cities is shown in (Fig. 3 and Table 2). The phase-wise percentage variation of the mean and standard deviation of pollutants for both cities during the lockdown is shown in Table 3. In Ankleshwar, the maximum decline was observed in NO_2_ (80%) during phase-2, while in Vapi the maximum drop was also in NO_2_ (91%) during phase-4 of lockdown. O_3_ increased by 192% and 310% in Ankleshwar and Vapi, respectively during phase-1. SO_2_ declined by − 67% during phase-1 and rose to − 28% during unlock 1.0 in Ankleshwar but it dropped to a maximum of 81% during phase-2, followed by an increase to more than 7% in comparison to the 2019 period during phase-4 in Vapi. PM_2.5_ ranged between − 36 and − 5% during phase-2 and phase-5, respectively in Ankleshwar. While in Vapi it ranged between − 48 and − 19% in phase-4 and phase-3, respectively. PM_10_ also showed a similar trend but it did not decline below 29% and 52% during phase-2 in Ankleshwar and Vapi, respectively. CO continued to increase in Ankleshwar from 30% in phase-1 to 150% in phase-5 (unlock 1.0), while in Vapi it had varied from 132% in phase-1 to − 38% in phase-3.

**Table 2:** Mean difference and standard deviation difference (rounded up for clarity) observed during different lockdown phases of 2020 in comparison to the same period in 2019 for Ankleshwar and Vapi cities.

Period	PM_2.5_		PM_10_		SO_2_		O_3_		CO		NO_2_	
	Mean diff (%)	SD diff (%)	Mean diff (%)	SD diff (%)	Mean diff (%)	SD diff (%)	Mean diff (%)	SD diff (%)	Mean diff (%)	SD diff (%)	Mean diff (%)	SD diff (%)
**Ankleshwar**												
Phase-1	− 10	− 46	− 19	− 30	− 67	− 75	192	113	30	− 35	− 67	− 67
Phase-2	− 36	− 74	− 29	− 50	− 63	− 38	98	20	74	− 20	− 80	− 71
Phase-3	− 6	131	5	231	− 54	− 51	44	35	84	100	− 50	33
Phase-4	− 26	− 3	− 27	67	− 30	− 16	− 31	86	91	243	− 37	5
Phase-5	− 5	335	24	11	− 28	123	44	− 1	150	− 99	27	35
**Vapi**												
Phase-1	− 25	− 24	− 51	− 54	− 32	− 16	310	215	132	194	− 43	− 38
Phase-2	− 40	− 59	− 52	− 56	− 81	− 30	83	64	− 22	225	− 53	− 4
Phase-3	− 19	223	− 21	62	− 12	− 39	− 53	− 79	− 38	− 56	− 55	81
Phase-4	− 48	4	− 38	− 1	7	− 45	− 59	− 68	− 18	− 50	− 91	− 70
Phase-5	− 35	260	− 36	135	− 24	− 9	− 36	30	− 20	− 105	− 86	− 71

**Table 3:** Variation air pollutant concentrations for pre-COVID19 (2019) and COVID19 (2020) years.

Pollutant	Phase 1 (Lockdown 1.0)				Phase 2 (Lockdown 2.0)				Phase 3 (Lockdown 3.0)				Phase 4 (Lockdown 4.0)				Phase 5 (Unlock 1.0)			
	Mean	Min	Max	SD	Mean	Min	Max	SD	Mean	Min	Max	SD	Mean	Min	Max	SD	Mean	Min	Max	SD
**ANKLESWAR 2019**																				
PM 2.5	48.670	1.870	86.410	18.750	50.731	33.470	171.000	30.138	31.881	25.780	38.800	3.995	32.943	23.070	41.660	4.751	26.482	23.870	30.060	2.076
PM 10	113.345	71.150	196.290	33.193	108.501	32.290	152.900	30.094	84.567	64.690	98.580	10.417	94.401	75.380	109.080	10.738	72.949	57.930	104.070	11.814
NO	18.015	9.230	26.670	5.690	24.689	10.610	32.590	5.892	8.139	6.680	9.710	0.823	6.732	3.790	9.340	1.762	4.882	3.130	9.890	1.748
SO_2_	28.995	17.500	79.250	15.707	21.488	13.370	39.670	7.089	12.310	7.550	16.990	3.354	10.843	6.400	16.220	3.246	9.448	6.140	14.870	2.990
CO	0.663	0.460	1.020	0.236	0.479	0.300	0.680	0.110	0.439	0.380	0.520	0.043	0.477	0.430	0.530	0.031	0.502	0.430	0.590	0.043
OZONE	17.113	6.920	27.880	5.245	19.184	7.640	29.740	5.582	20.302	10.090	30.680	6.317	41.095	34.490	48.970	4.418	25.217	11.990	41.370	7.538
**ANKLESWAR 2020**																				
PM 2.5	43.694	28.540	78.540	10.112	32.439	21.610	57.280	7.735	30.107	20.230	47.270	8.919	24.424	16.970	37.110	4.615	25.259	19.140	46.790	9.012
PM 10	91.413	49.380	148.190	24.285	77.031	54.560	113.200	14.617	88.775	50.010	154.230	33.151	68.518	49.130	125.690	18.244	50.338	35.800	86.550	12.659
NO	5.849	3.400	10.410	1.887	4.894	2.540	9.150	1.669	4.066	2.980	5.500	1.042	4.166	2.640	9.000	2.028	6.219	3.150	29.760	6.582
SO_2_	9.434	4.370	21.900	3.972	7.986	2.890	21.250	4.383	6.138	3.360	9.760	1.930	7.567	4.050	14.340	2.711	6.646	2.440	28.480	6.439
CO	0.861	0.390	1.170	0.157	0.826	0.670	1.020	0.088	0.782	0.690	0.870	0.067	0.880	0.540	1.290	0.249	1.251	0.750	5.380	1.252
OZONE	50.023	24.530	68.360	11.155	37.945	27.080	51.960	6.544	29.428	14.270	39.050	8.082	27.741	16.970	44.140	8.762	36.327	21.160	46.690	7.189
**VAPI 2019**																				
PM 2.5	72.230	42.780	132.080	26.584	61.099	33.020	108.700	19.346	39.774	34.370	45.260	3.308	35.334	21.940	54.810	9.504	23.391	18.120	27.700	2.975
PM 10	139.818	84.190	238.000	53.067	131.908	82.210	204.250	36.470	86.347	68.580	111.150	13.842	79.172	48.040	113.300	18.160	51.563	41.650	59.060	5.076
NO	23.396	13.730	32.330	6.203	31.212	15.780	54.420	9.916	25.991	12.640	39.550	6.787	23.904	17.190	32.440	5.066	29.682	17.260	44.210	7.869
SO_2_	19.142	12.890	25.580	4.045	32.505	19.910	76.030	12.663	18.468	11.810	21.470	2.487	21.428	15.480	26.590	2.745	17.591	9.020	26.150	3.876
CO	0.878	0.540	1.240	0.178	0.714	0.420	1.170	0.241	0.813	0.580	1.190	0.169	0.658	0.440	1.020	0.208	0.734	0.430	1.020	0.188
OZONE	6.216	3.130	15.950	3.785	16.535	3.580	30.890	9.011	28.796	12.410	42.300	10.593	26.639	17.400	45.920	9.252	15.531	10.270	21.530	3.146
**VAPI 2020**																				
PM 2.5	43.694	28.540	78.540	10.112	32.439	21.610	57.280	7.735	30.107	20.230	47.270	8.919	24.424	16.970	37.110	4.615	25.259	19.140	46.790	9.012
PM 10	91.413	49.380	148.190	24.285	77.031	54.560	113.200	14.617	88.775	50.010	154.230	33.151	68.518	49.130	125.690	18.244	50.338	35.800	86.550	12.659
NO	5.849	3.400	10.410	1.887	4.894	2.540	9.150	1.669	4.066	2.980	5.500	1.042	4.166	2.640	9.000	2.028	6.219	3.150	29.760	6.582
SO_2_	9.434	4.370	21.900	3.972	7.986	2.890	21.250	4.383	6.138	3.360	9.760	1.930	7.567	4.050	14.340	2.711	6.646	2.440	28.480	6.439
CO	0.861	0.390	1.170	0.157	0.826	0.670	1.020	0.088	0.782	0.690	0.870	0.067	0.880	0.540	1.290	0.249	1.251	0.750	5.380	1.252
OZONE	50.023	24.530	68.360	11.155	37.945	27.080	51.960	6.544	29.428	14.270	39.050	8.082	27.741	16.970	44.140	8.762	36.327	21.160	46.690	7.189

All pollutants are in units of μg/m^3^ and CO is expressed in units of mg/m^3^.

*Min* minimum, *Max* maximum, *SD* standard deviation.

**Figure 4:**
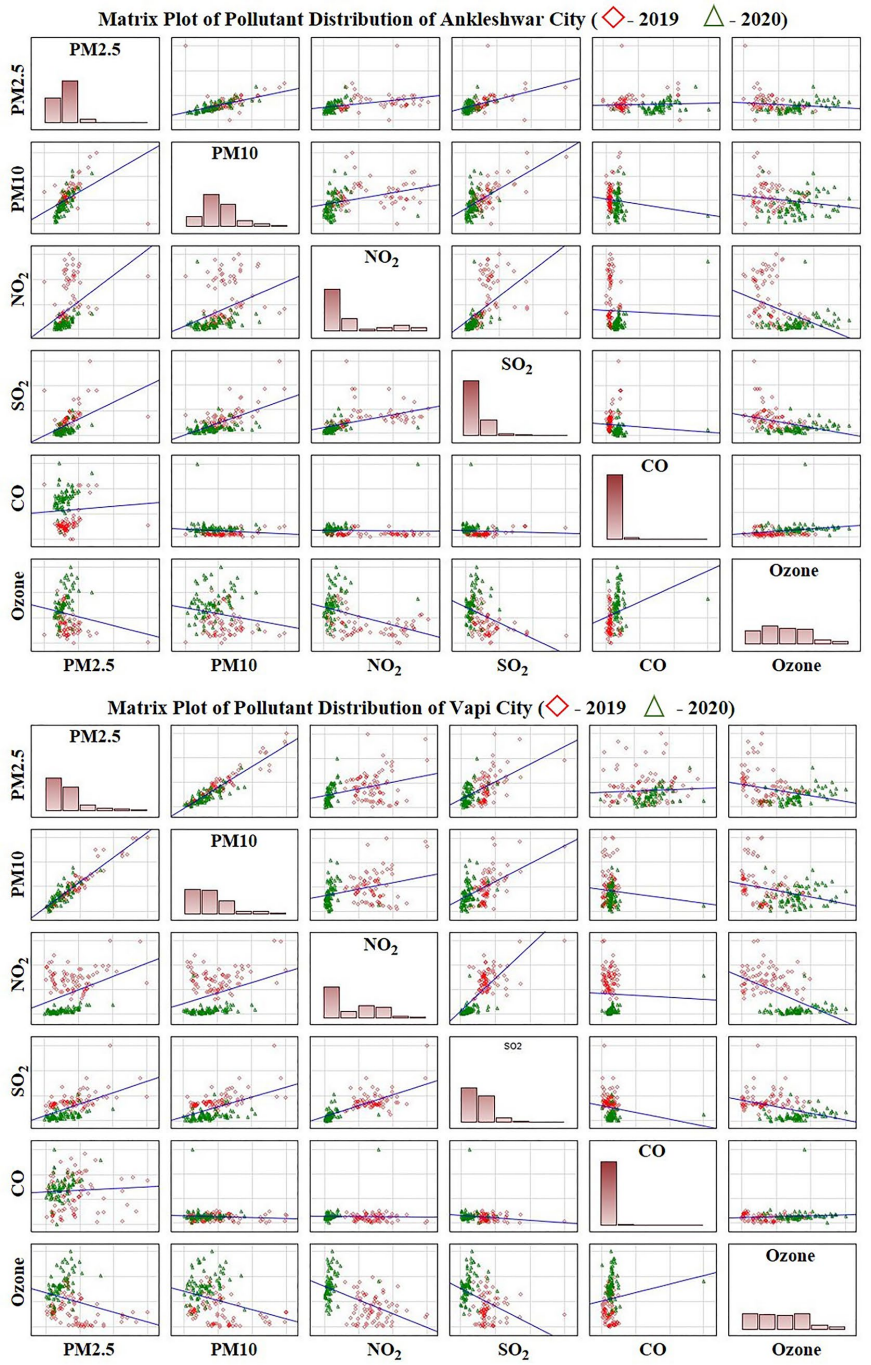
Correlation matrix scatter plot of the air pollutants (The Diagonal Bar Diagrams are the density plots fo various pollutant variables showing the distribution of data).

**Figure 5:**
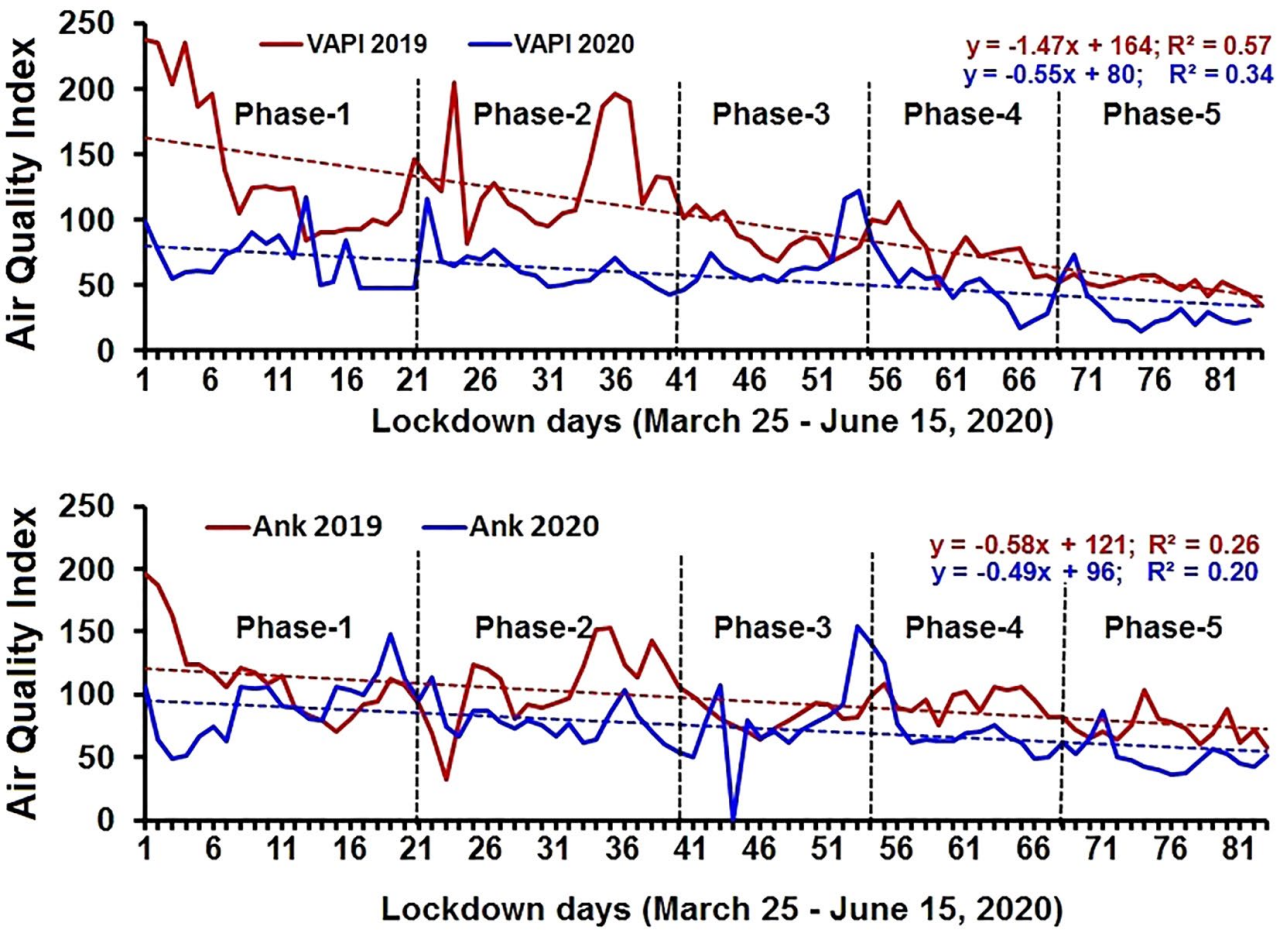
Linear regression of AQI for Vapi and Ankleshwar for 2019 and 2020.

The linear correlations of metadata of each pollutant and linear regression between pollutant concentrations and lockdown days are shown in the matrix plot (Fig. 4) and regression plot (Fig. 5) respectively. A declining trend is observed in particulate matter (PM_2.5_ and PM_10_), SO_2,_ and NO_2_ levels during the COVID-19 lockdown period, as compared to the pre-COVID-19 year (Table 1). The O_3_ concentration increased during the lockdown period compared to the pre-lockdown due to a decrease in NO_2_ content. The distribution of CO shows a variable trend. A scatter plot matrix (Fig. 4), a grid (or matrix) of scatter plots, a graphical equivalent of the correlation matrix, is used to assess air pollutant variable data and to visualize the bivariate relationships between combinations of variables of pre-COVID19 (2019) to COVID19 (2020) in both cities. Each scatter plot in the matrix visualizes the relationship between a pair of variables, allowing many relationships between several pairs of all air pollutant variables to be explored at once. The Matrix of scatter plot (Fig. 4) clearly shows the variable distribution of the pollutant variables from pre-COVID19 (2019) and COVID19 lockdown period (2020) in all combination of scatter plots. It is also clearly observable from Matrix plot, a positive correlation between particulate matter (PM_2.5_ and PM_10_) with NO_2_, SO_2,_ and CO. O_3_ shows a negative correlation with particulate matter (PM_2.5_ and PM_10_), SO_2_ and NO_2._

Further, we also note that the linear regression analysis exhibited a negative correlation between the daily AQI and the growing number of lockdown days (Fig. 5). The main reason behind the negative correlation could be the meteorological conditions prevailing in the region. In South Gujarat region during 2020 and 2019 a southerly gentle breeze with a speed of 2–4 m s^**‒**1^ prevailed^23^ combined with the closure of transport and industries for a longer continuous period in comparison to the similar pre-COVID-19 period^41^. We believe that the prevalence of consistent wind speed and direction in the South Gujarat region during the 2020 lockdown and similar period of 2019 (along with different restrictions imposed during the lockdown in 2020) has helped in reducing pollutant levels.

The results of the present study corroborates with other recent similar studies conducted in various cities across the globe (USA^3^; China^12,13,15^; Brazil^16^; Italy^42^ India^23^).

## Discussion

Environmental degradation due to anthropogenic pollution has become a chronic problem the world over. In India, haphazard development has led to various problems such as land degradation and air–water quality degradation, mainly in urban areas. Air pollution has become a severe problem in various metropolitan cities and industrialized centers across the country. Incomplete combustion of fossil fuels by vehicles and industrial operations^42^, and improper disposal of anthropogenic waste are the root causes of the rapid increases in air pollution. In March 2020, the COVID-19 pandemic led to a nationwide lockdown to control the spread of infection. The total stretch of various phases of lockdown was 68 days, in which the restrictions were eased subsequently. Although temporarily, the long stretch of restrictions on economic activities provided an opportunity for the environment to heal itself from the continuous exploitation by human activities^8^.

It is evident from the recent studies that reductions in most of the pollutants was observed all over India during the lockdown period. In a study across 12 cities, located in different spatial segments Indo-Gangetic Plain (IGP), showed a substantial decrease (35%) of PM2.5 concentrations across the cities located in IGP after implementation of lockdown^43^. In Saurashtra and South Gujarat regions in Gujarat state, reductions up to 30–84% in NO_2_ concentration was observed, while O_3_ increased by 16–48% due to reduction in NO_2_. The average decrease in AQI values of 58% was mainly observed in industrial cities such as Ahmedabad, Gandhinagar, Jamnagar, and Rajkot^32^. The atmospheric pollution level (NO2, PM2.5, and PM10) in Ahmedabad city also showed a significant improvement during the study period, implying a positive response of COVID-19 imposed lockdown on the environmental front^44^. In Delhi, the pollutant level came down to its 5-year low during the first week of lockdown phase-1, where PM_2.5_ concentration dropped to 42 μg/m^3^ (similar to the values observed in March 2016)^45^. AQI reduction in Delhi was 49% compared to the previous year, thus improvement of about 60% was mainly observed in the industrial and transport hub^33^. During the lockdown period, reduction in PM_2.5_ among all the pollutants was maximum in Gaya, Kanpur, Nagpur, and Kolkata^23^. The results of the study^46^ of variation in ambient air quality during COVID-19 lockdown in Chandigarh showed significant reductions in all air pollutants during the first and second phases of the Lockdown. The concentration of PM10, PM2.5, NO2 and SO2 reduced by 55%, 49%, 60% and 19%, and 44%, 37%, 78% and 39% for Delhi and Mumbai, respectively, during post-lockdown phase leading to a significant improvement in air quality^47^. The reduction in mean concentration from the pre-lockdown phase to during lockdown of the main air pollutants is observed in Kolkatta City^48^. In a similar study^49^, on 16 cities designated as Hotspot region covering almost two thirds of India, also reported a significant reduction in the observed (mean) levels of PM10, PM2.5 and NO2 concentration during the lockdown period from March 25 to April 25.

The present study to assess the effect of COVID-19 lockdown in reducing air pollution in the two industrial cities viz., Ankleshwar and Vapi, Gujarat, India substantiate earlier studies. In comparison to a similar period in 2019, Ankleshwar observed a reduction in PM_10_ (primarily emitted by vehicles) and PM_2.5_ (caused by dust, ash, etc.)^39^ concentrations during phase-1, while in Vapi the concentrations were reduced to almost half of those in the previous year (Fig. 3). The most plausible reason being the total restrictions on vehicular movements and industrial activities during the lockdown period. Similar drastically decreasing trends were observed for SO_2_ in Ankleshwar (highest reduction among all phases) produced by transportation and oil refineries^50,51^. Vapi also recorded a one-third reduction in its mean SO_2_ values.

Reduction in NO_2_, (which is emitted by heavy vehicles^52^ during all the phases consequently spiked the ozone concentration in both the cities (reduction in NO_x_ can increase ozone due to nonlinear relationships just above the ground level^53^). CO, which is commonly produced by incomplete combustion of carbon-containing fuels^54^, showed rising trends in Ankleshwar during all the phases but dropped in Vapi.

Although rainfall, a significant determinant which helps to lower the pollutant levels occurred negligibly during the study period in both the cities, which implies that, in the absence of good amount of rainfall, which is a vital factor of pollution reduction, the difference in the trends is a result of continuous operation of the majority of pharmaceutical industries establishments in Ankleshwar during the lockdown. At the same time, Vapi showed a reduction in CO (except in phase-4 and phase-5) due to the non-operation of the majority of industries during the lockdown phase. Phase-2 lockdown continued for 14 days during which the PM_2.5_ along with PM_10_, SO_2_, and NO_2_ showed decreasing trends, whereas SO_2_ significantly dropped in all phases in both the cities (Table 2).

During phase-3, with restrictions eased, economic activities were restarted in a gradual manner, which led to the increasing trend in the pollutants. In Ankleshwar, during phase-4 (lockdown) and phase-5 (unlock-1), PM_2.5_ and PM_10_ mean concentrations exhibited an increasing trend due to increased vehicular movement and industrial operations, but Vapi still showed significant decreasing trends compared to 2019. The continuous operations of pharmaceutical industries during the lockdown phases, primarily the large-scale production ‘Hydroxychloroquine' which is considered effective in COVID-19 treatment at Zydus Cadila, Vital Pharma and Mangalam Drugs and Organics located in Ankleshwar and Vapi, and its transportation to the seaports, is probably one of the main reasons for increases in vehicle-related pollutants (NOx, SO_2,_ CO) since mid-phase-3 due to its global demand. Which can further be verified from the statement “In Ankleshwar, Vatva and Vapi the major sectors contributing to air pollution were transport, industries and power plants” given by a senior Gujarat Pollution Control Board official (John, April 7, 2020). “Construction, road dust re-suspension, and residential activities also contributed to pollution. In addition landfill fires, operation of DG sets, cooking at restaurants also added to pollution”^55^.

Concurrently, it is evident from the negative correlation between air quality index values of Ankleshwar and Vapi and COVID-19 lockdown days (Fig. 5) that the majority of air pollutants decreased since March 25, 2020 as the lockdown period got extended in a phased manner. The most obvious reason was the shutting down of the industrial and transport sector since the lockdown phase-1 started and as the days progressed, pollutants were subsequently flushed out^40^.

However, different trends of some pollutants (minimal increment during later phases) are probably due to two primary causes. First, the diversity in the industrial setup and decline in the number of the on-road vehicle^3,56^. Ankleshwar hosts the majority of pharmaceutical manufacturing plants, while Vapi houses more heterogeneous industries, such as pharmaceutical, petrochemical, etc. This difference indirectly influences the emission of different pollutants (as per the raw material used for the processing) and the transportation of the finished products to the market.

**Figure 6:**
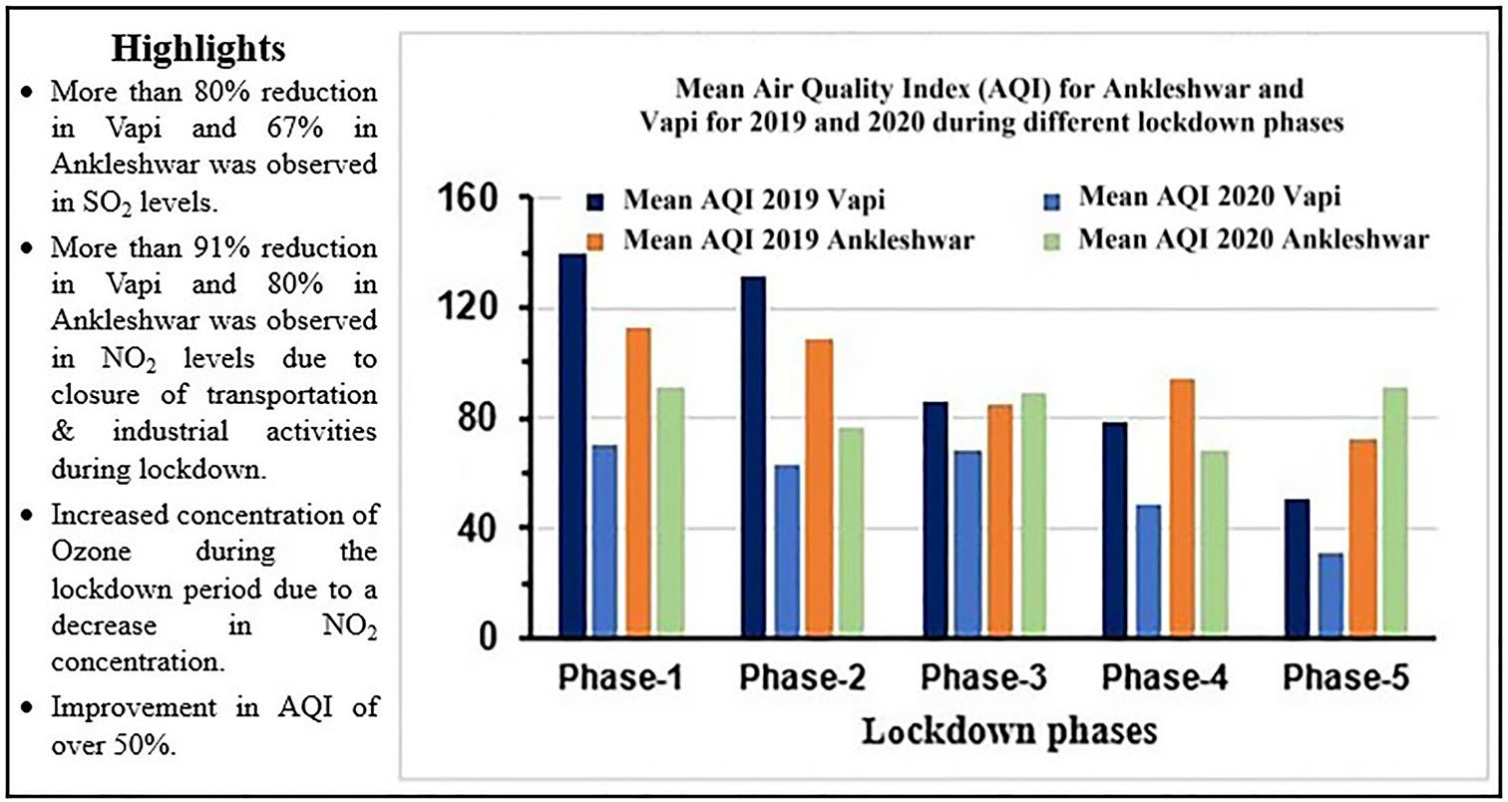
Mean Air Quality Index for Ankleshwar and Vapi for 2019 and 2020 during different lockdown phases.

Secondly, and more importantly, the proximity of Vapi to the Arabian coast (8 km) is significantly lesser than Ankleshwar (38 km), which also plays a vital role in mixing up and higher fluctuation of pollutant range (due to sea-land breeze) in comparison to Ankleshwar. These causes suggest the differences in the rate of reduction and increment of the concentration of pollutants in the two cities. Figure 6 presents a summary of highlights and the variation in the Air Quality Index (AQI) corresponding to different lockdown phases.

## Conclusion

It is undoubtedly evidenced from this study and the others in several cities^57^, that the lockdown measures imposed to contain the spread of COVID-19 infection was found to be very effective resulting in a positive impact during the Pandemic as a blessing in disguise. It not only restricted the spread of infection rate, but also has given a scope to realize the restoration ability of environment and health with reduced ambient air pollutants levels leading to improved air quality.

The bold decision to impose strict lockdown measures by the Government of India despite economic losses, on the positive front, these measures brought significant improvement in air quality. The present study takes into consideration of the air pollutant observation of all the 5 phases of lockdown period, in contrast to the earlier studies from different parts of the country, that are restricted to only the earlier phases of lockdown period, supports the improved air quality due to lockdown measures. This study highlights the air quality index data of two industrialized cities Ankleshwar and Vapi to determine the trends of different pollutants during all the lockdown phases of COVID-19 in India. Both cities have been classified as critically polluted in Gujarat during the last decade^58^. However, the present study showed a drastic overall reduction of pollutants in both the cities. The results revealed different patterns of gradual to a rapid reduction in most of the pollutant concentrations additionally an increment in O_3_ concentration due to drastic reduction in NO_2_ by as much as 80.18%. Increases in other pollutants were also observed as the restrictions were eased during phase-4 and unlock 1.

The different lockdown phases were differentiated based on subsequent relaxation in the norms to restart economic activities. The world’s largest lockdown event has provided an actual example instead of modeled scenarios to determine how the pollutants can fluctuate due to different economic restrictions. Due to fear of infection, individuals embraced the restrictions imposed by the lockdown. Thus, it allowed us to show the levels of reduction possible to curb pollution levels.

Although the meteorological conditions (specifically rainfall) could also play a role in bringing down the levels of air pollutions, however, the impact of rainfall is in very minimal in the present study area as no rainfall is reported during the studied duration. The impact of the meteorological conditions cannot be ignored and should be considered in the future for understanding the long term trends.

It is obvious that there is clear reduction in the pollutants levels due to COVID-19 related lockdown improving the air quality in most of parts of the earth as observed from the earlier studies and from the present study. The system imposed was certainly harsh for the economy. However, a modified mode of various reservations for the economy could be used to manage pollution levels on a case-to-case basis. The findings of the present study certainly offers potential scope to plan air pollution reduction strategies. For policymakers, the need for the hour is to acknowledge the role of lockdown in curbing air pollution and not to lose the lead unintentionally achieved during this period against rising air pollution to critical levels. It is hoped that the present situation will open the perspective of humans in understanding deleterious effects of anthropogenic activities.

## Data Availability

The Daily CPCB AQI data for more than 200 Indian stations as available open-source at https://app.cpcbccr.com/ccr/#/caaqm-dashboard-all/caaqm-landing.
